# Intravascular Myopericytoma: A Case Report

**DOI:** 10.7759/cureus.28581

**Published:** 2022-08-30

**Authors:** Mohamed B Mohamed, Mohamed Idris, Sameh Bibawy

**Affiliations:** 1 Plastic and Reconstructive Surgery, Hull University Teaching Hospital, Hull, GBR; 2 Internal Medicine, Hull University Teaching Hospital, Hull, GBR

**Keywords:** tumor, intravascular, immunohistochemistry, connective and soft tissue neoplasms, myopericytoma

## Abstract

Myopericytoma is a rare tumor that arises from perivascular myoid cells. Intravascular myopericytoma is an exceptionally rare subtype with a small number of cases reported. Here, we describe the case of a 31-year-old woman who presented with a lump on the dorsum of the right foot for nine months. Imaging indicated that the lesion is in close proximity to the dorsalis pedis vessels. Following surgical excision, the histological analysis revealed a benign neoplasm arising within a vein wall with features of vascular and pericytic differentiation. When using immunohistochemistry, the blood vessels were highlighted by the cluster of differentiation (CD) 31 and smooth muscle actin (SMA) with negative staining for pancytokeratins. These features led to the diagnosis of intravascular myopericytoma.

## Introduction

Myopericytoma (MPC) is considered a soft tissue tumor with a benign nature and was recently discovered [1]. The World Health Organization endorsed the terminology in 2002 to define a lesion that consists of a concentric perivascular growth pattern of myoid-like cells that vary in shape from oval to spindle [2]. The great majority of lesions appear to originate in the dermis and subcutaneous tissue with a clear preference for the extremities [3]. Although there have been reports of malignant instances and recurrence, these tumors are typically benign [4]. It is believed to be a distinct perivascular myoid cell (myopericyte) neoplasm even though it resembles glomus tumor, angiolipoma, angioleiomyoma, and myofibroma morphologically [5].

Intravascular myopericytoma (IVMP), first described by McMenamin and Calonje in 2002 [3], is a unique and uncommon histological variation, with only six cases reported in the English literature. Clinically, the intravascular subtype of MPC typically has a painful subcutaneous lump despite the fact that most MPCs are rarely painful. The IVMP exhibits the same histological characteristics as MPC and manifests as a distinct intravenous solid mass occupying a previously distended large vascular lumen [6]. In this paper, we describe a case of intravascular myopericytoma affecting the dorsum of the foot.

## Case presentation

A 31-year-old woman presented with a nine-month history of a lump on the dorsum of her right foot. It started small and gradually increased in size with pain and discomfort. Her medical history had no related information or any evidence of prior trauma. Clinical examination revealed a 2 cm cystic mobile swelling with negative Tinnel's sign.

**Figure 1:**
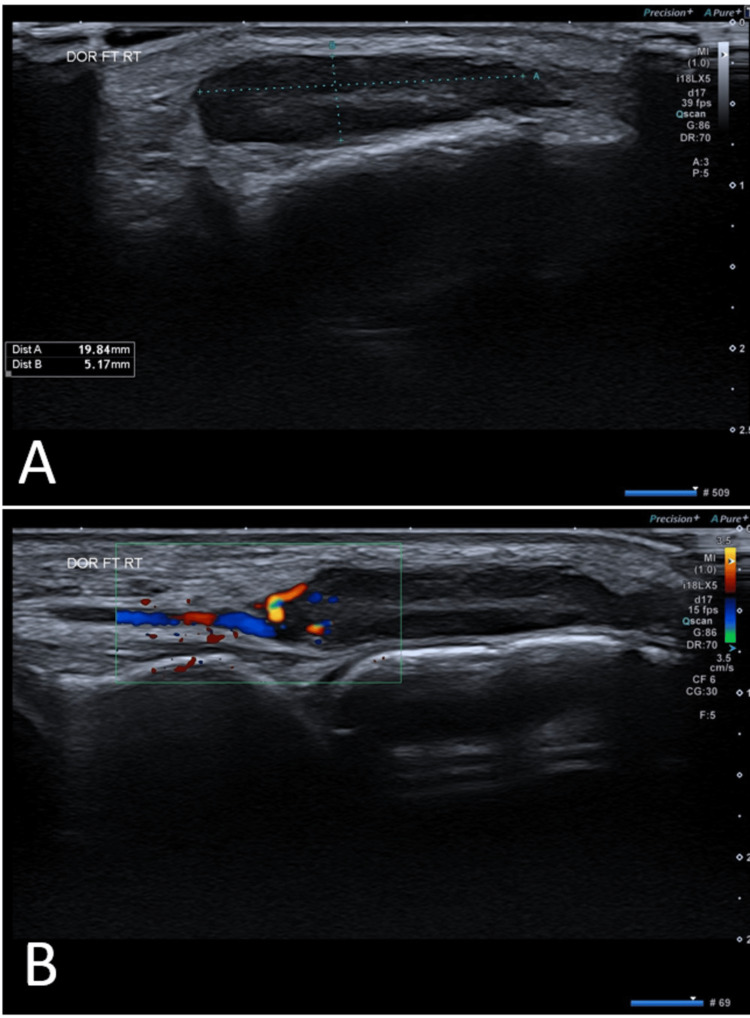
Ultrasound showing a heterogeneous well-circumscribed mass on the dorsum of the right foot measuring 19 x 5 x 4 mm

**Figure 2:**
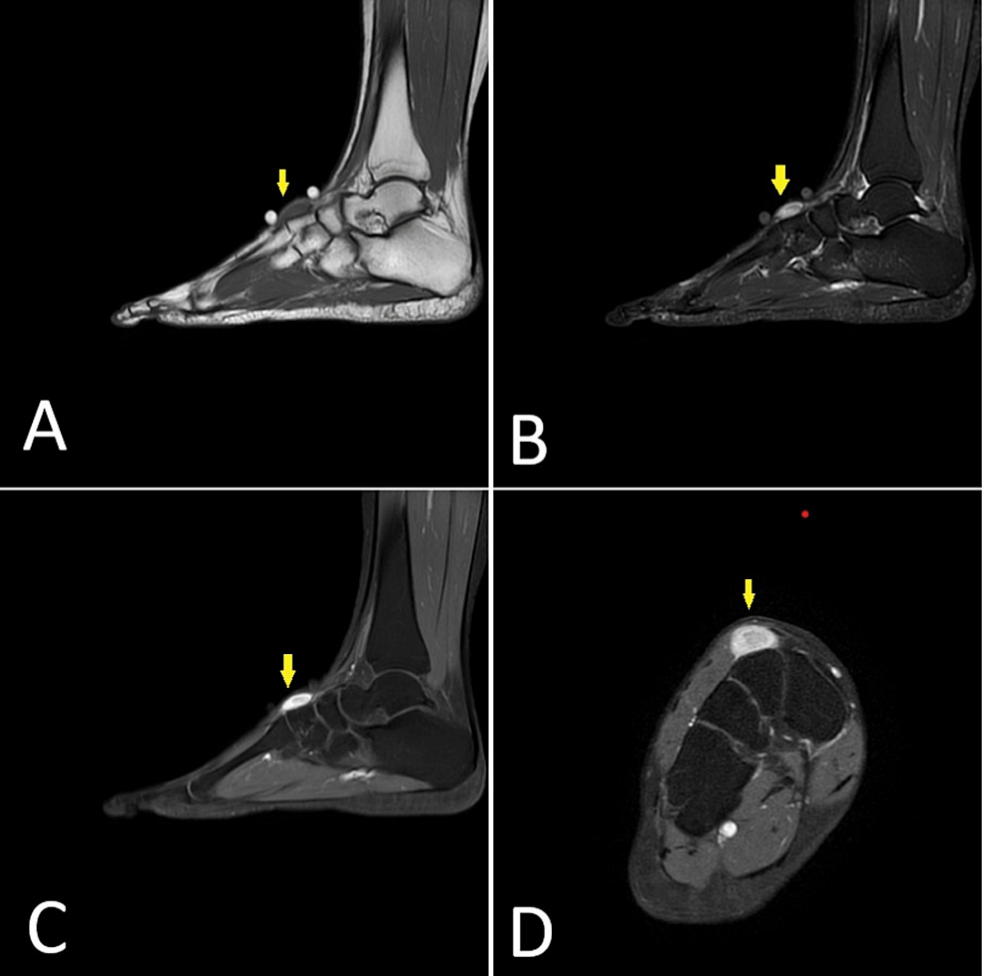
MRI of the foot and ankle showing the lesion on the dorsum of the right foot A: Sagittal T1  FSE showing the lesion (arrow) with a low signal, B: Sagittal FSE STIR where the lesion (arrow) shows a high signal, C: Water–sagittal T1 FSE post Gadolinium with the lesion (arrow) enhances post gadolinium, D: Water–axial T1 FSE post Gadolinium with the lesion (arrow) enhances post gadolinium FSE: Fast spin echo, STIR: Short tau inversion recovery, MRI: Magnetic resonance imaging

An ultrasound (US) scan showed a heterogeneous, well-circumscribed mass of 19 x 5 x 4 mm of uncertain etiology (Figure 1). Further imaging with magnetic resonance imaging (MRI) revealed an elliptical mass measuring approximately 2.6 x 0.83 cm, a low signal on T1, a high signal on the fat saturation sequences, and the lesion enhanced following gadolinium (Figure 2). Additionally, it was seen that the lesion lies over the dorsum of the intermediate cuneiform and does not communicate with a joint.

The patient was discussed in the sarcoma multidisciplinary team (MDT) and the outcome was a mass adjacent to the dorsalis pedis artery with no sinister features. The lesion was completely excised and sent for histology.

Macroscopically, it appeared as a firm pale grey tissue measuring 22 x 15 x 7.0 mm. Microscopically, the section showed a well-circumscribed nodular lesion composed of small-caliber thin-walled blood vessels intermixed with a round to oval monomorphic cells, the stroma is fibrous with keloidal changes, and focal thrombosis is noted in the center of the lesion. By immunohistochemistry, the blood vessels were highlighted by cluster of differentiation (CD) 31 and smooth muscle actin (SMA), which were also positive in single scattered cells between the blood vessels, and there was negative staining for pancytokeratins. These findings were in keeping with a benign neoplasm arising within a vein wall with features of vascular and pericytic differentiation and led to the conclusion that the patient had intravascular myopericytoma.

## Discussion

An MPC is a benign tumor characterized by a myoid/pericytic line of differentiation. Myopericytes were first described in a report by Dictor et al. in 1992 [7]. The report was about a tumor on the thyroid gland of a five-year-old boy. Most MPCs are not painful, but the intravascular subtype often presents as a painful mass under the skin. The intravascular position of the tumor and the formation of a thrombus may have contributed to the pain [3]. To our knowledge, there are six reported cases of IVMP: two in the thigh [3,6], oral mucosa [8], leg [9], infraorbital [10], and heel of the foot [11]. However, McMenamin and Calonje [3] proposed that the first case of IVMP was diagnosed as an intravascular angioleiomyoma [12], and the instance they presented was essentially the second.

It has been observed that a typical MPC consists of monomorphic myoid-like cells with an oval to spindle configuration that have conspicuous multi-layered concentric proliferation around lesion blood vessels [5]. The prominent, concentric perivascular growth of myoid tumor cells is the characteristic histologic hallmark of myopericytoma. In immunohistochemistry, these cells are usually positive for smooth muscle actin (SMA), h-caldesmon, and partially for desmin, but lack expression for S100, CD34, cytokeratin, and human melanoma black 45. However, unlike myopericytoma, IVMP is negative for desmin.

In our case, neuroma was the first differential diagnosis due to the painful nature and position of the swelling, perivascular myoma, angioleiomyoma, angiosarcoma, and hemangiopericytoma were all considered because of the proximity of the lesion to the dorsalis pedis on imaging. Other reactive or neoplastic lesions that might be intravascular should be considered as part of the differential diagnosis for IVMP such as glomus tumor [13], which lacks sections of both myofibroma-like spindle cells and rich eosinophilic cytoplasm, papillary endothelial hyperplasia [14], that manifests as multiple small papillary formations surrounded by a single layer of flattened endothelium, which resembles an organizing thrombus, pyogenic granuloma [15], which display a lobular pattern of capillary and venule development, nodular fasciitis [16], which is made up of immature, spindly myofibroblasts that closely resemble those in granulation tissue or tissue cultures, and leiomyomatosis [17].

Imaging is typically ineffective for evaluating the vascular nature of IVMP. However, US and MRI can be utilized to characterize the morphology and for surgical planning [12].

## Conclusions

In summary, we have reported a case of intravascular myopericytoma in the dorsum of the foot. Intravascular myopericytoma is a benign, rare, recently discovered soft-tissue tumor that grows inside blood vessels. Furthermore, the similarity of IVMP to other intravascular lesions makes immunohistochemistry tests essential.
